# Supplementary material for: Library adaptors with integrated reference controls improve the accuracy and reliability of nanopore sequencing
Source: Nat Commun. 2022 Oct 28;13:6437. doi: 10.1038/s41467-022-34028-8 (PMC9616880; doi:10.1038/s41467-022-34028-8)
Supplement: Supplementary file 1 — Supplementary Information [file 41467_2022_34028_MOESM1_ESM.pdf]

Library adaptors with integrated reference controls improves accuracy and reliability of nanopore sequencing.

Helen M. Gunter[1], Scott E. Youlten[2], Bindu Swapna Madala[2], Andre L. M. Reis[2], Igor Stevanovski[2], Ted Wong[2], Sarah Kummerfield[2,4], Ira W. Deveson[2,4], Nadia S. Santini[3], Esteban Marcellin[1] & Tim R. Mercer[1,2*]

SUPPLEMENTARY MATERIALS

Supplementary Figures – See page 1-10

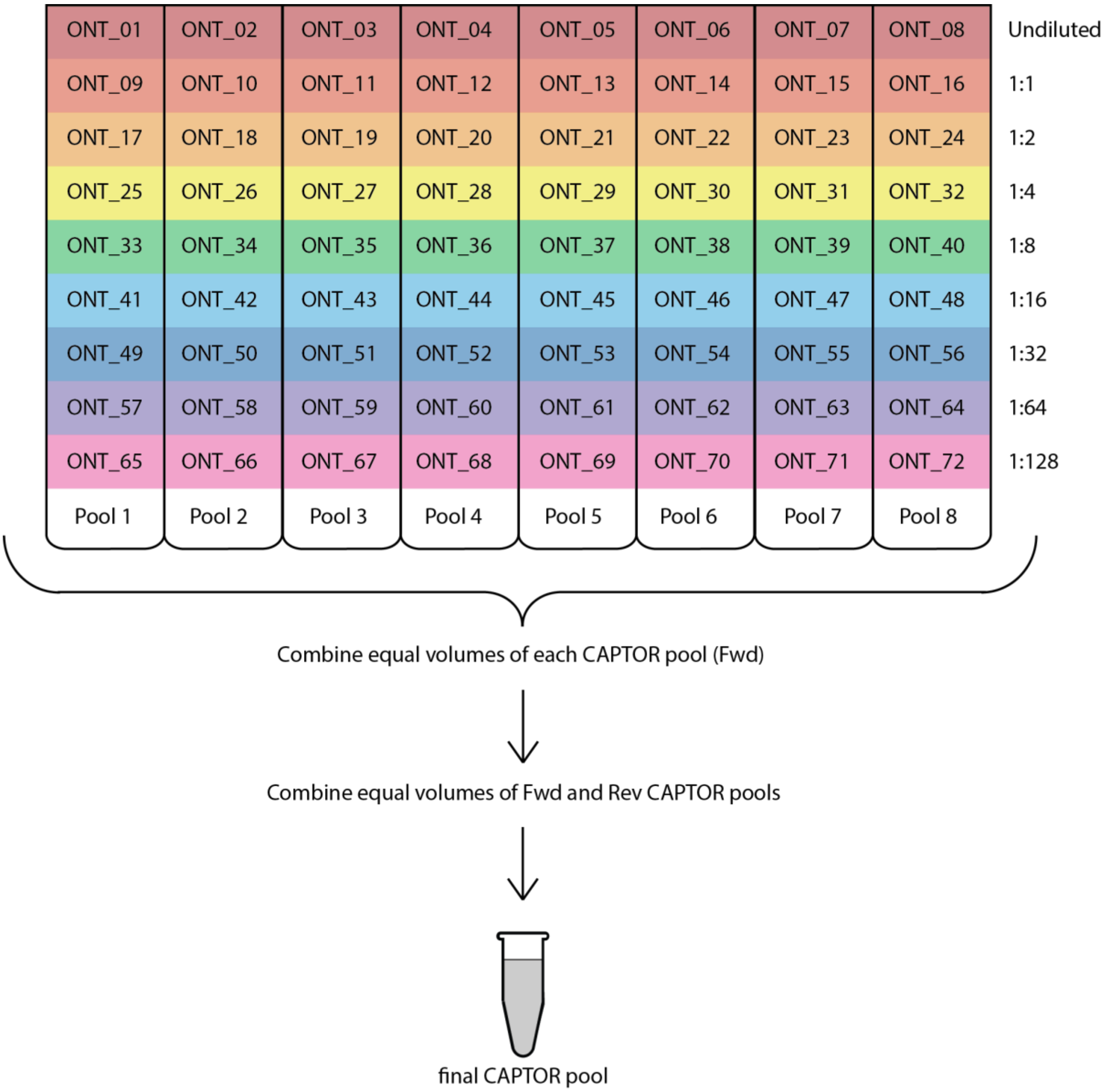

Supplementary Fig. 1 Pooling strategy for CAPTORs. We prepared pools where the 72 Forward and Reverse CAPTORs (each labelled ONT_01 – ONT_72) were inputted at staggered concentrations, ranging from undiluted to 1:128. The Forward and Reverse CAPTOR pools were combined in an equimolar ratio to form our final CAPTOR master mixture, that was used in our library preparations.

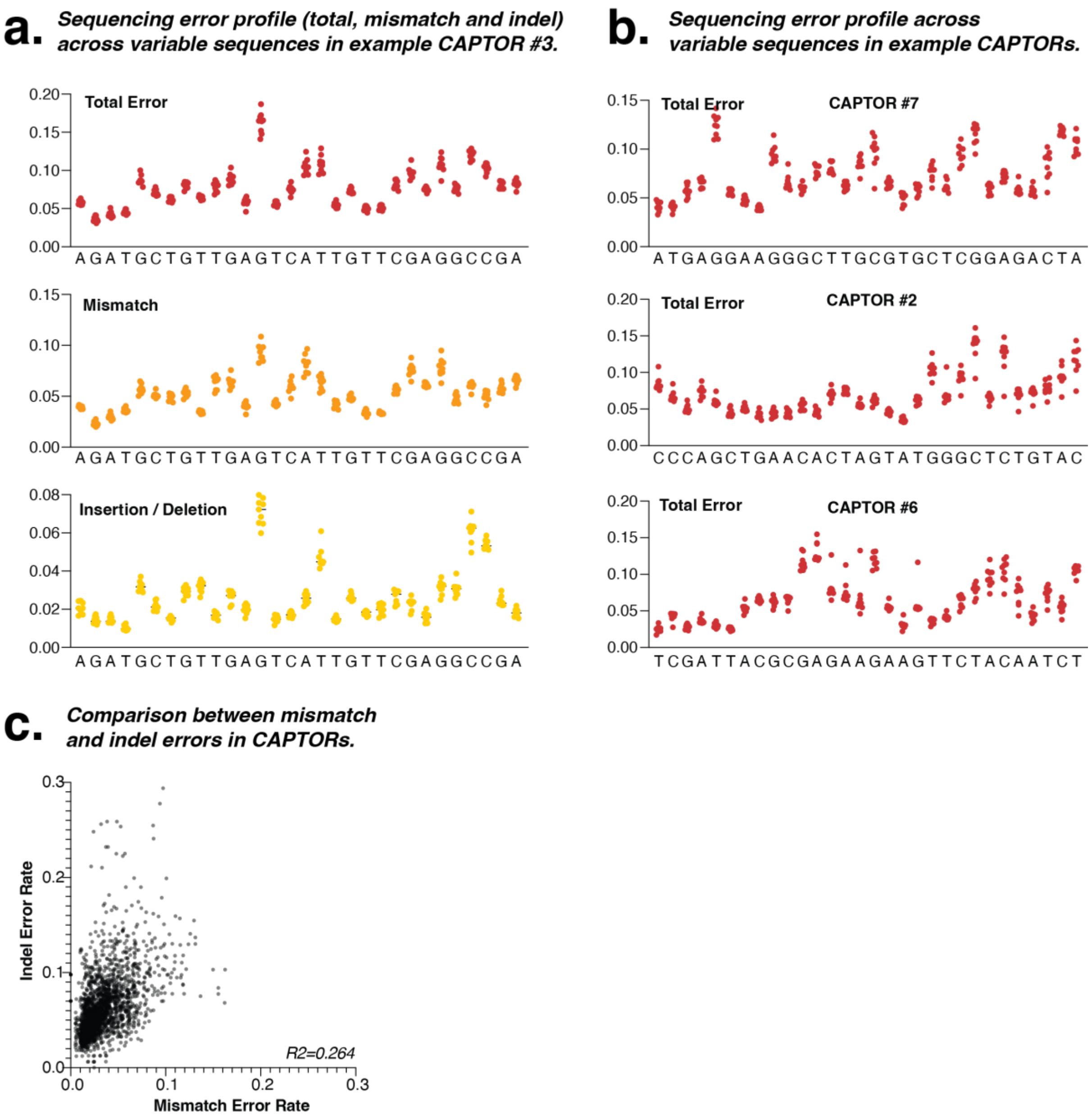

Supplementary Fig 2 a Histograms show per-nucleotide sequencing error across variable sequence of an example CAPTOR from replicate libraries (n=9). The total (upper panel), mismatch (middle panel) and insertion/deletion (indel; lower panel) error profiles are shown. **b** Histograms show per-nucleotide sequencing error across variable sequences of three CAPTOR examples (7,2 and 6). **c** Scatter plot compares rate of mismatch and insertion/deletion errors for each individual 6-mer within a single library.

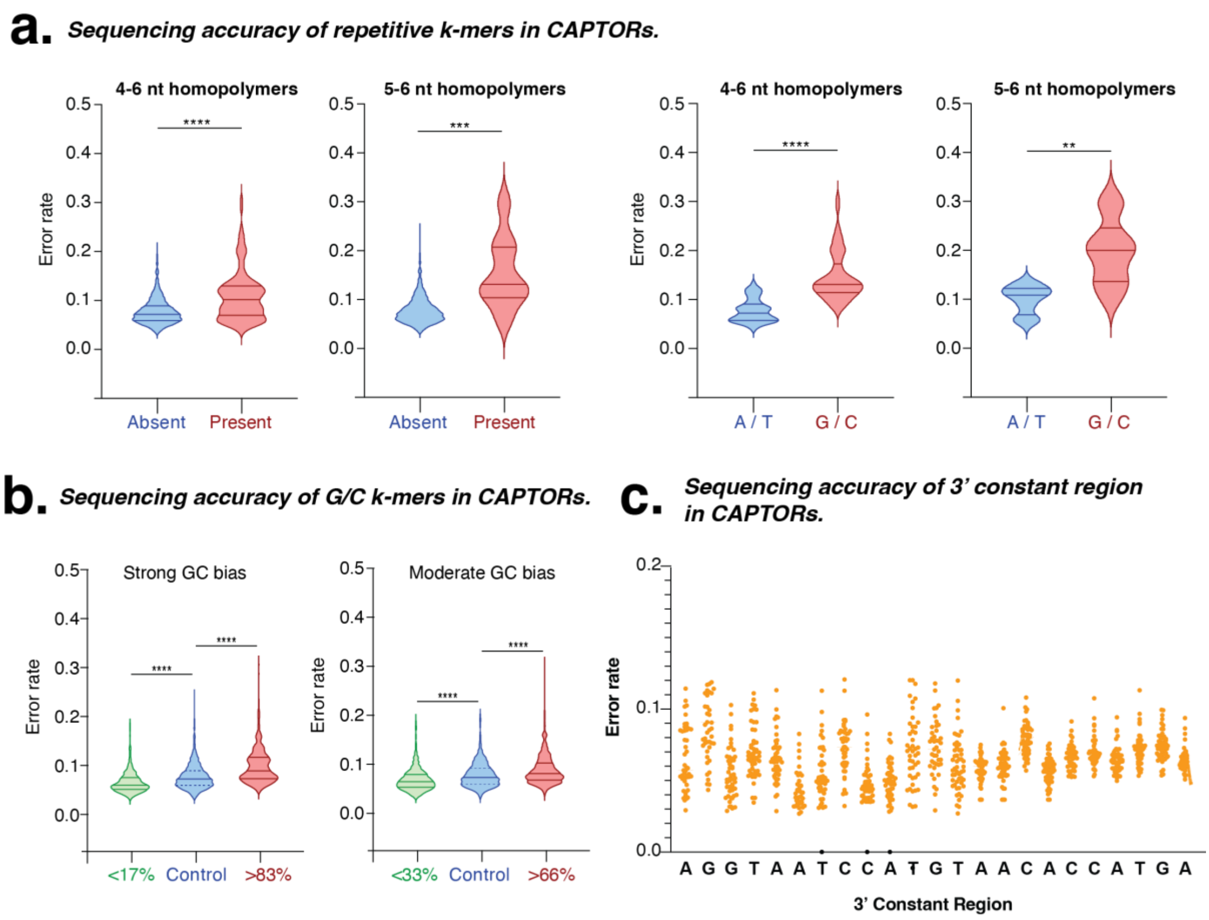

Supplementary Fig. 3 Sequencing accuracy at 'difficult' sequences. Violin plots show the impact of the GC-rich and low-complexity sequences on error rate. **a** Violin plots show error rates in k-mers with homopolymer stretches of 4-6 or 5-6 nucleotides (nt), compared to k-mers that lack homopolymer stretches. Asterisks indicate results of Welch's two tailed t-test without multiple testing correction. We demonstrate that the presence of homopolymers and their GC content impacts sequencing error rate. Presence vs absence of 4-6 nt homopolymers in CAPTORs results in significantly different error rates p<0.0001 (****), 95% CI = 0.02263 to 0.04078; similar to presence vs absence of 5-6 nt homopolymers p=0.0003 (***), 95% CI = -0.1118 to -0.03999. Additionally, CAPTORs that include 4-6 nt homopolymers comprised of A/T vs G/C show significantly different error rates p<0.0001 (****), 95% CI = 0.05492 to 0.08334; similar to 5-6 nt homopolymers comprised of A/T vs G/C p=0.0011 (**), 95% CI = 0.04740 to 0.1488. **b** Violin plots show error rate at GC-rich or GC-poor k-mers. Asterisks indicate results of Welch's ANOVA without multiple testing correction. For strong GC bias p<0.0001 (****). For moderate GC bias P<0.0001 (****). **c** Scatter plot shows the variation in error profile across the 3' constant region of CAPTORs.

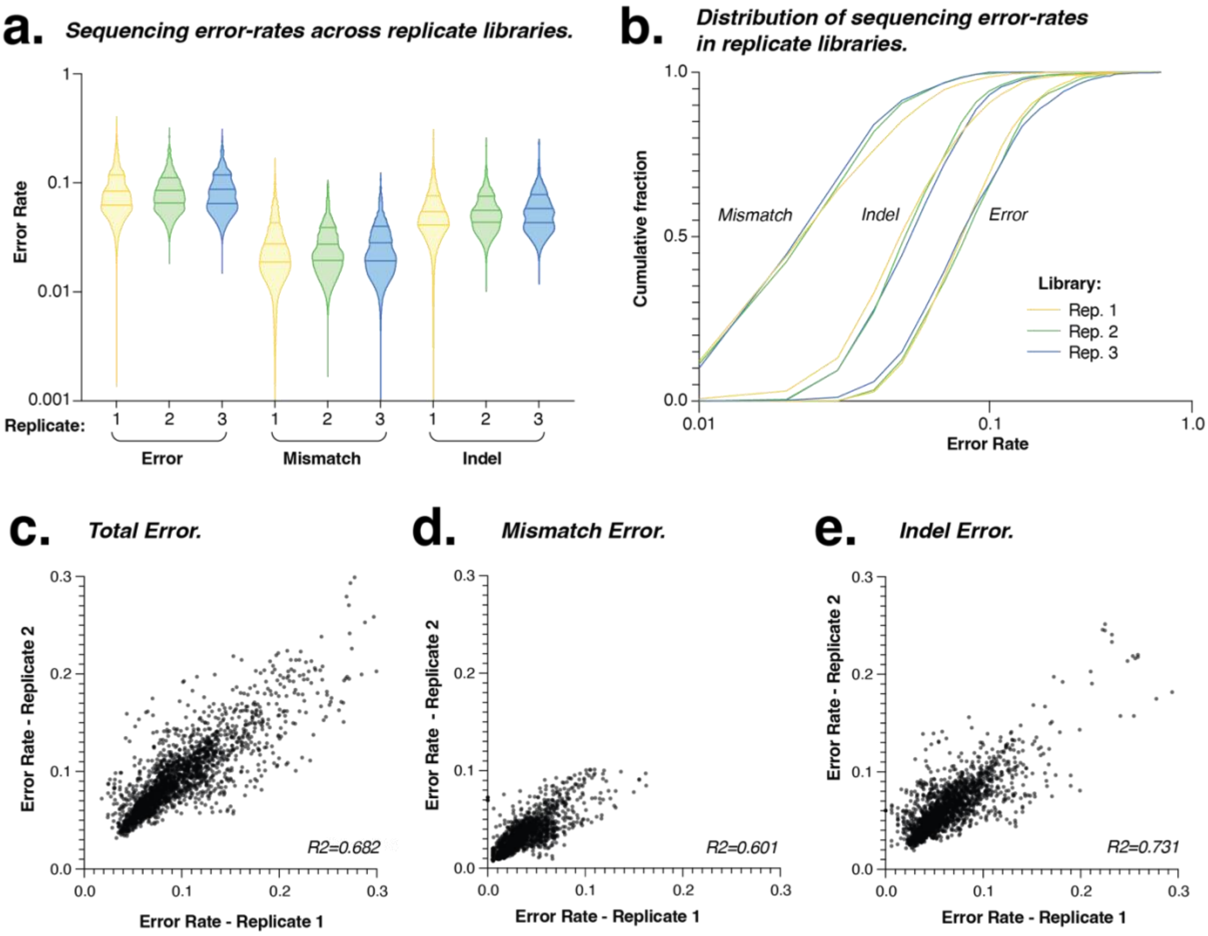

Supplementary Fig. 4 a Violin plot shows the distribution of sequencing (total, mismatch and indel) errors between three replicate libraries (Rep. 1-3). **b** Cumulative distribution plot illustrates the distribution of sequencing errors across three replicate libraries. **c-e** Scatter plots show pairwise comparison between two replicate libraries for total sequencing errors**.**

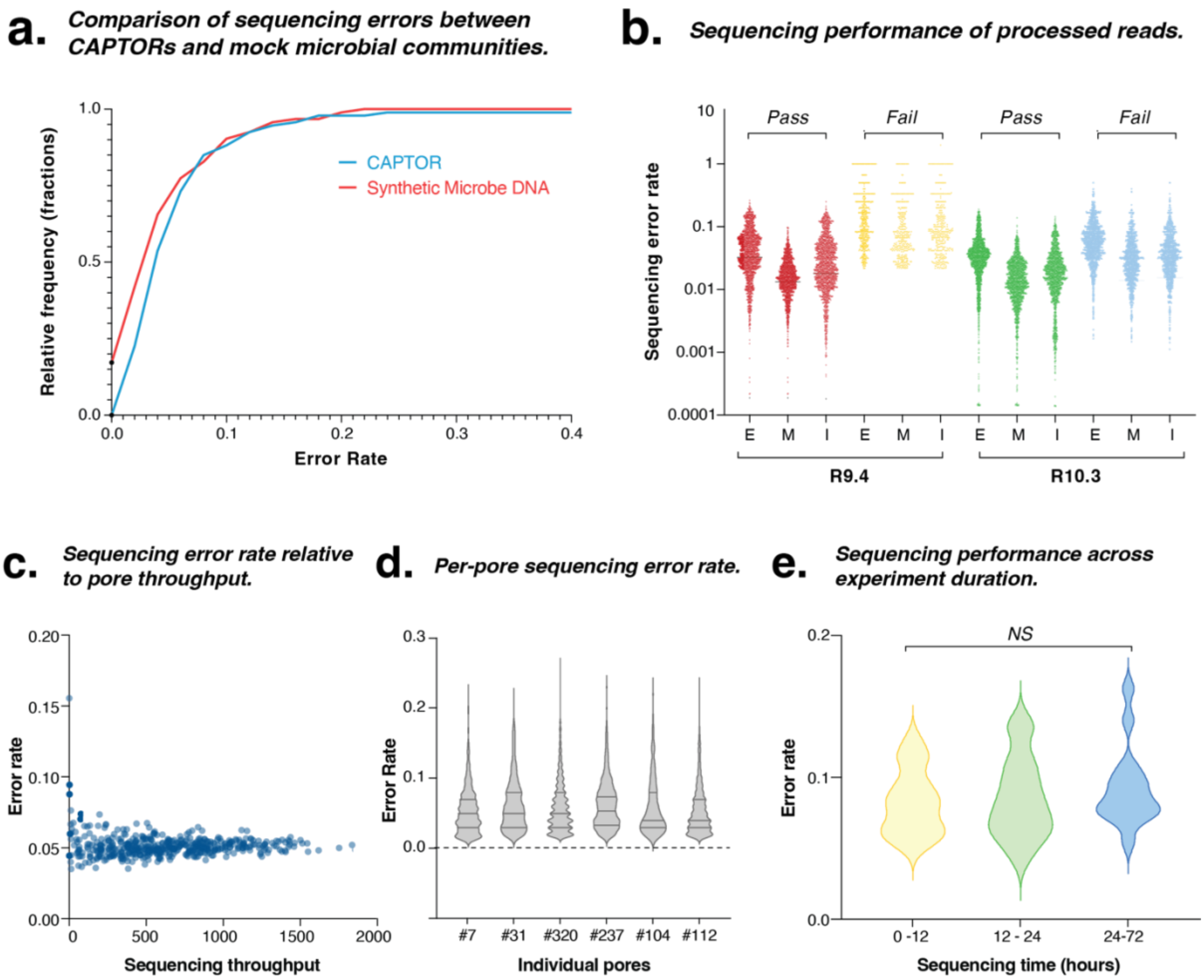

Supplementary Fig. 5 a Cumulative frequency distribution plot compares the sequencing error rate of CAPTORs to error rate of accompanying synthetic microbial sequences. **b** Violin plot illustrates the mean sequencing accuracy (for total Errors, Mismatch and Indels) in CAPTORs ligated to reads that 'pass' or 'fail' according to ONT classification. **c** Scatter-plot compares the sequencing error rates of individual pores compared to sequencing throughput. **d** Violin plot shows the distribution of sequence errors for individual pore examples. **e** Violin plot shows the distribution of sequencing errors for pores that continued to generate sequence throughout the duration of the sequencing experiment (up to 12, 12 to 24, and 24 to 72 hours). 2-way ANOVA indicates no significant difference (NS) in error across the 3 different time periods (p=0.1308).

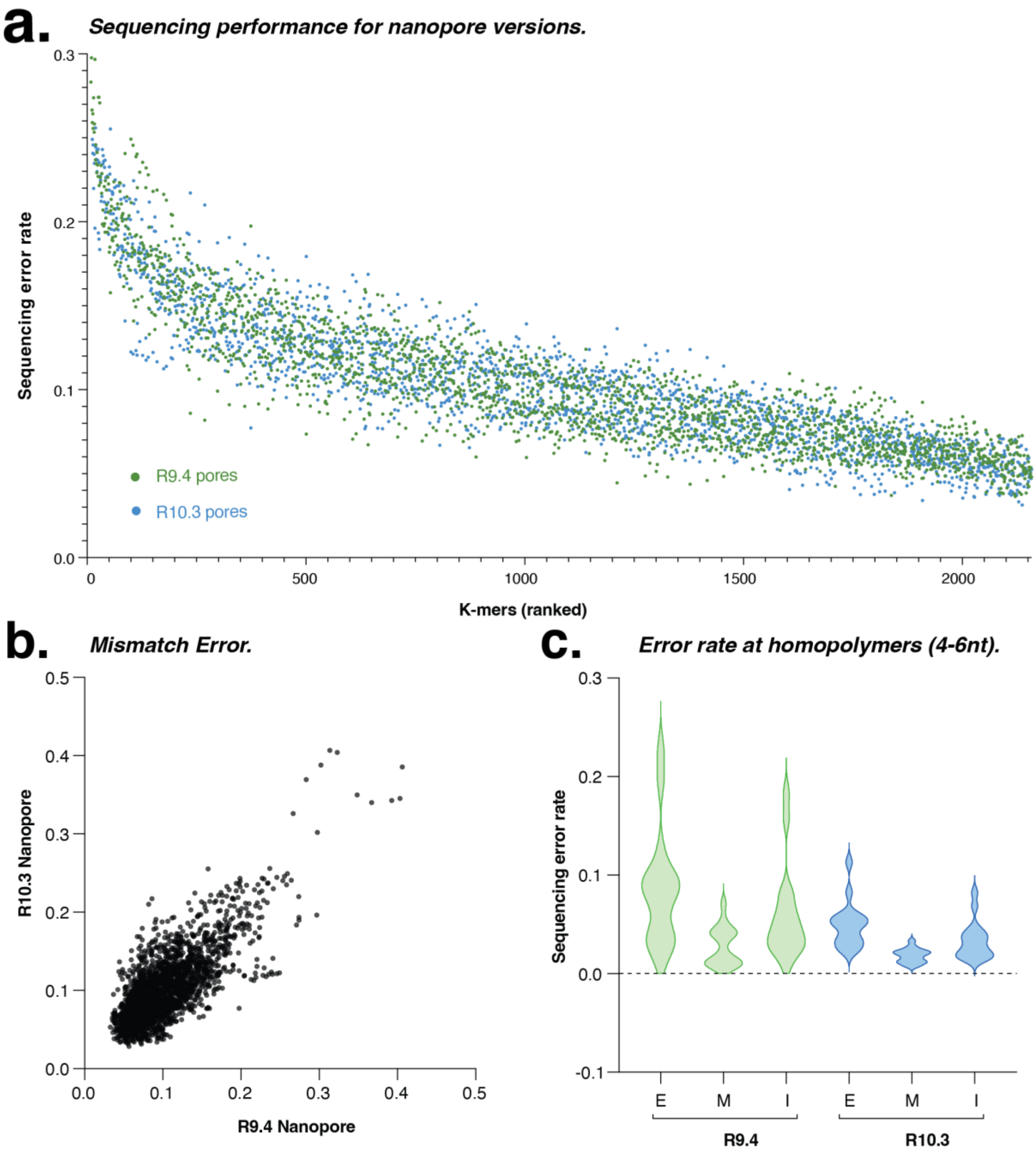

Supplementary Fig. 6 a Scatter plot shows the sequencing error profiles for 6-mers (ranked by total error rate) for the R9.4 and R10.3 nanopore versions. **b** Scatter plot compares the sequencing error for 6-mers within CAPTORs measured by either R9.4 or R10.3 nanopore versions. **c** Violin plot shows the distribution of sequencing errors (total Error, Mismatch and Indel) at homopolymers of >4 nt (nucleotides) for the R9.4 and R10.3 nanopore versions.

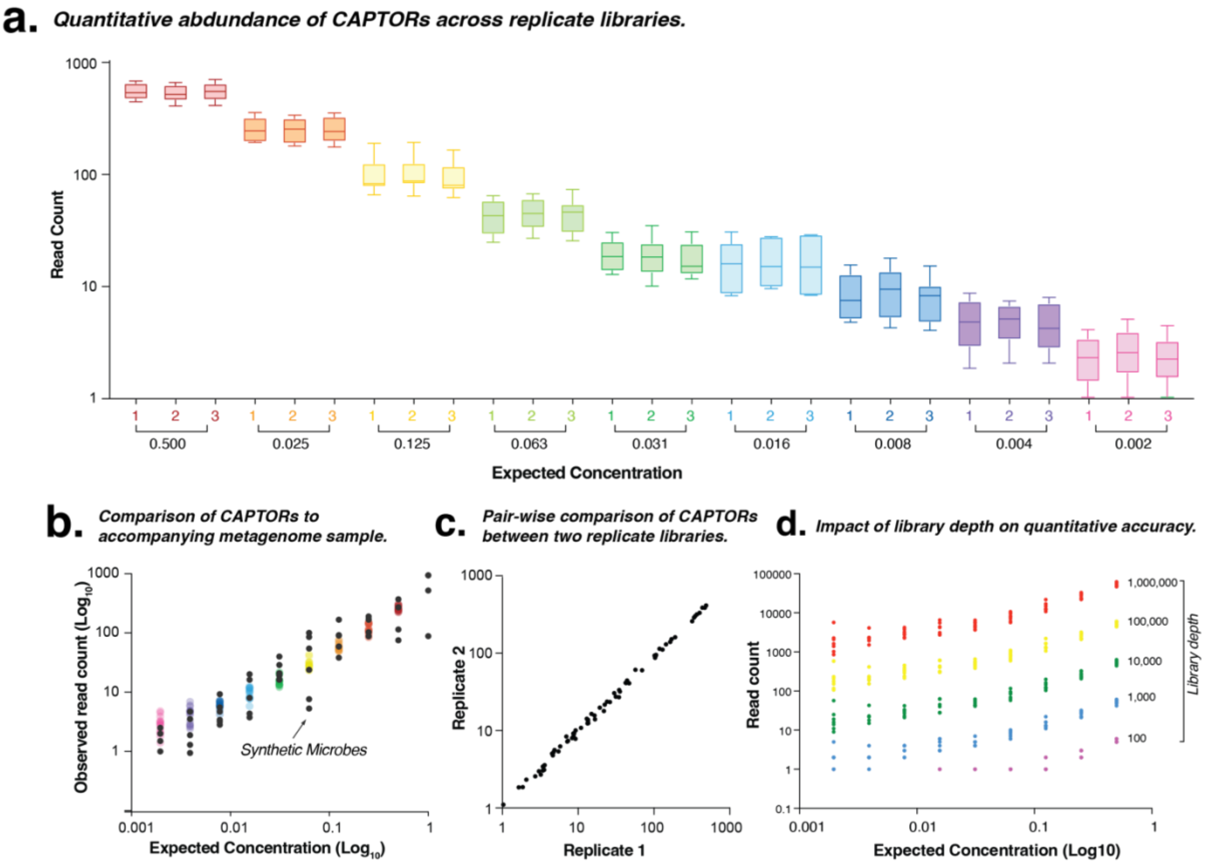

Supplementary Fig. 7 a Box-whisker plots show the quantification of CAPTORs that form a staggered reference ladder across three technical replicate libraries, prepared in separate laboratories (1-3). Quartiles of box plots are indicated (25%, 50% and 75%). **b** Scatter plot compares the Co-efficient of Variation (COV%) to the expected concentration for different CAPTORs, demonstrating a lower COV% for libraries present at higher concentrations. **c** Scatter-plot compares the quantification of matched CAPTORs between two technical replicate libraries. **d** Scatter plots illustrate the CAPTOR reference ladders following the subsampling of libraries at decreasing sequencing depths.

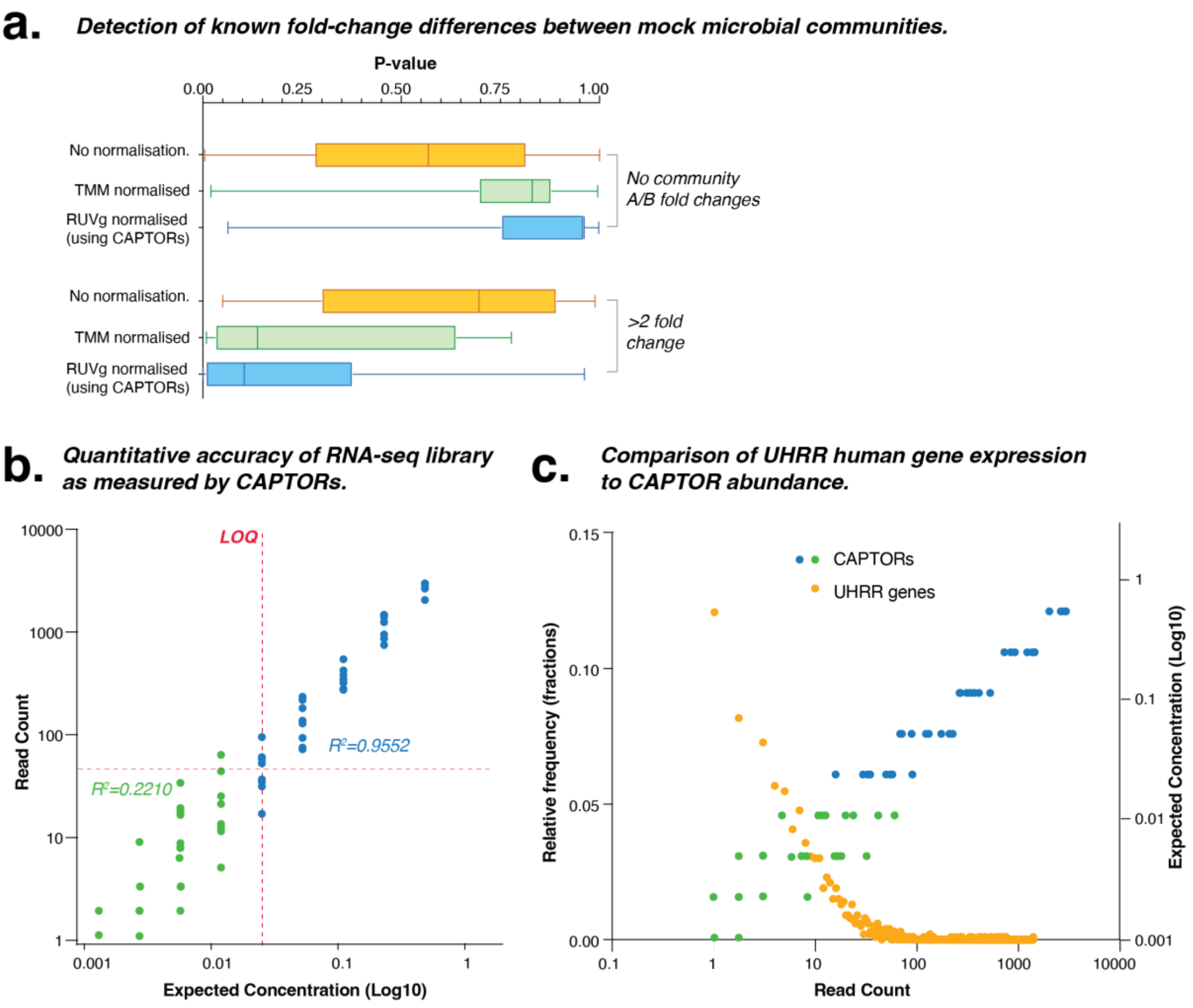

Supplementary Fig. 8 a Histogram shows the significance of detecting known fold-changes between mock microbial communities following no normalisation, Trimmed Mean of M-values (TMM) normalisation and Removal of Unwanted Variation (RUVg) normalisation with CAPTORs. Data derived from three technical replicates, prepared in separate laboratories. Quartiles of box plots are indicated (25%, 50% and 75%). **b** Scatter-plot shows the quantification of CAPTORs within RNA sequencing library. Limit of quantification (LOQ, dashed line), determined by segmental linear regression, is indicated. **c** Scatter-plot shows the quantification of human genes in the Universal Human Reference RNA (UHRR) sample compared to the CAPTORs that fall both above and below the LOQ.

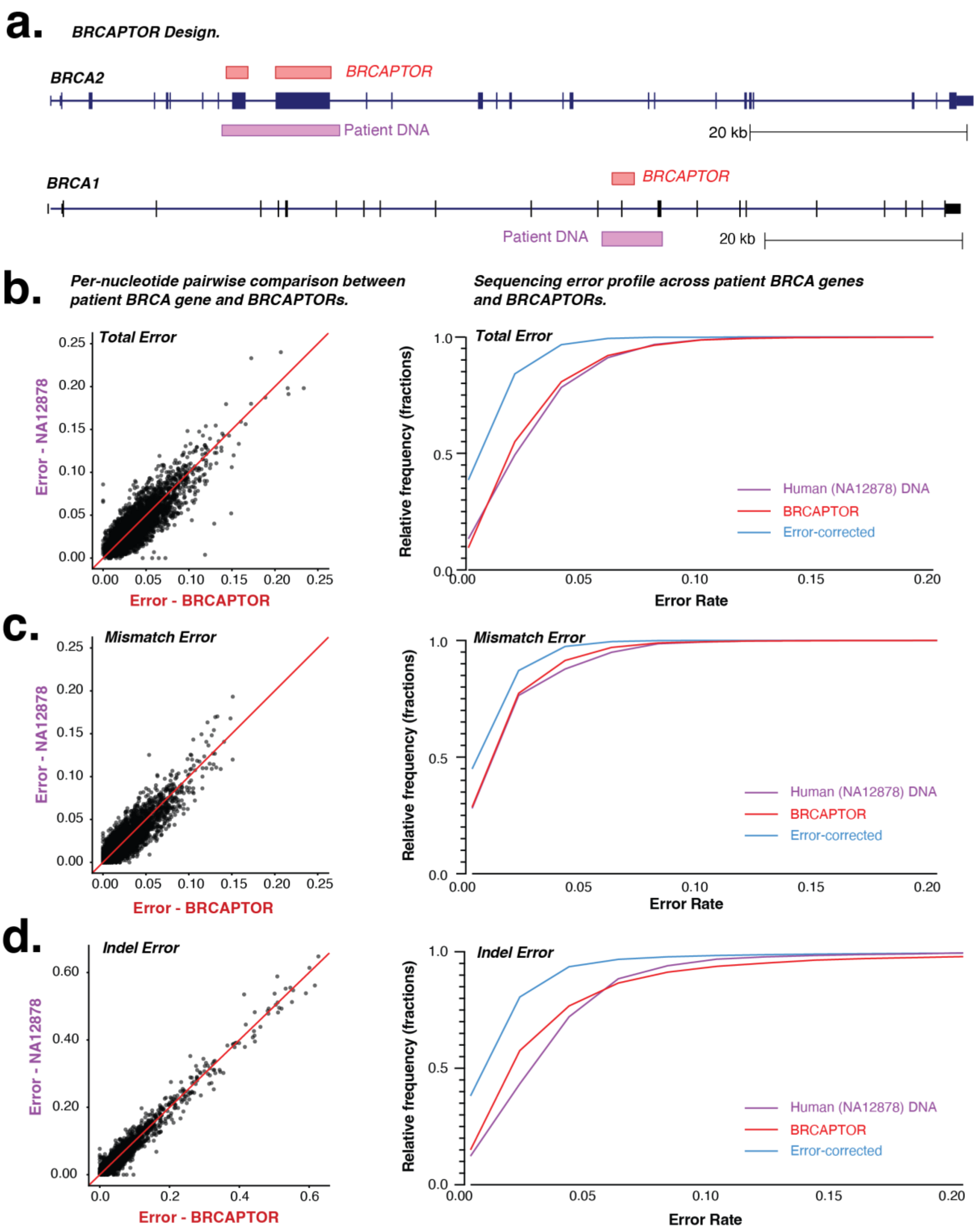

Supplementary Fig. 9 a Genome browser shows the design of *BRCAPTORs* that represent clinically-important sequencing within *BRCA1* and *BRCA2* genes. **(b-d)** Scatter plots (left) show the per-nucleotide correlation between **b** total, **c** mismatch, **d** and indel errors in BRCAPTORs and NA12878 human DNA sample. Accompanying cumulative distribution plots (right) show sequencing error across NA12878 human DNA, BRCAPTORs and following error-correction strategy.

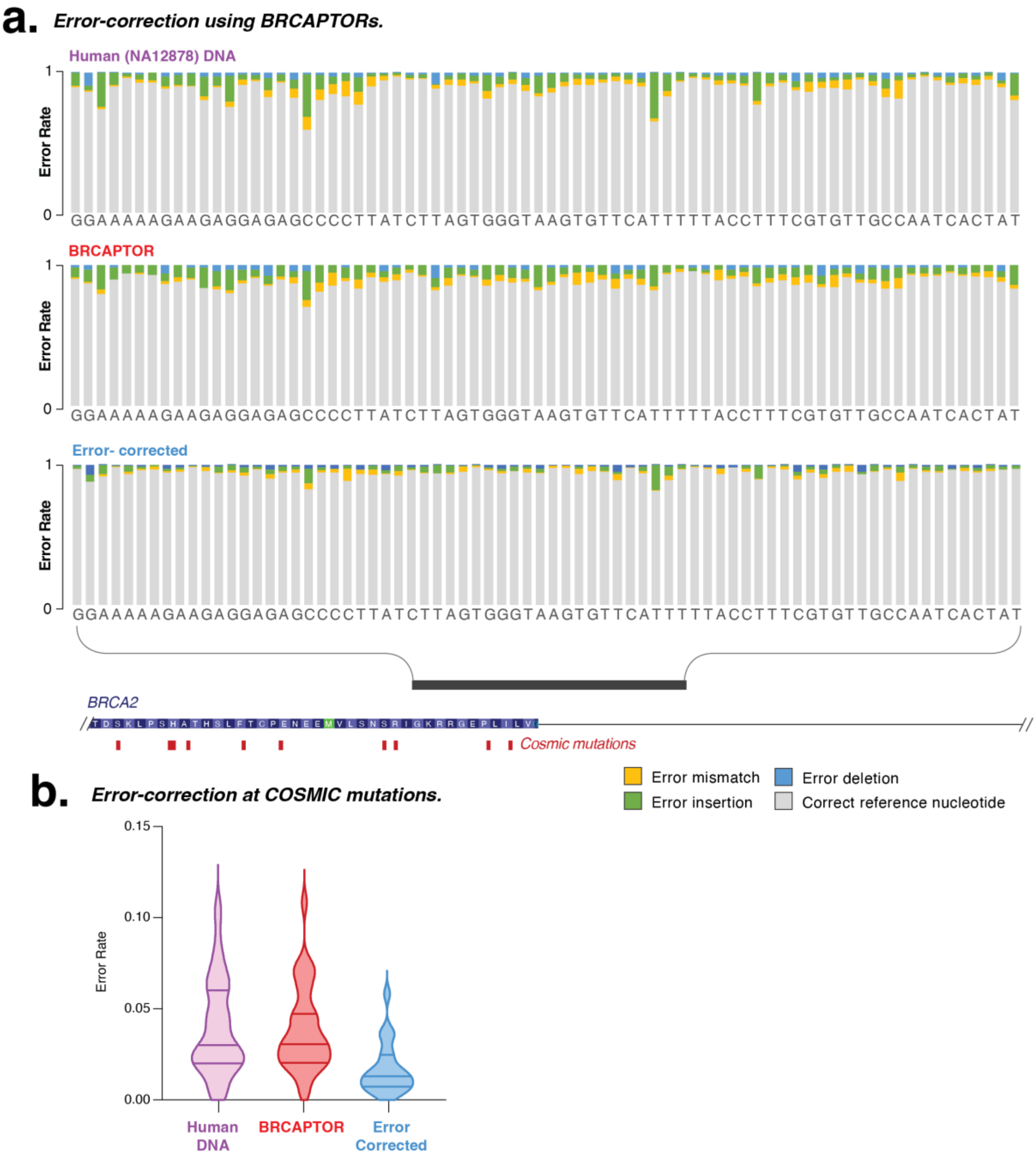

Supplementary Fig. 10 a Per-nucleotide profile shows the sequencing error rate for corresponding BRCA2 sequencing within NA12878 human DNA sample, *BRCAPTOR* and error-corrected sequence. Genome browser view showing *BRCA2* gene and cancer-associated mutations listed in the Catalogue of Somatic Mutations In Cancer (COSMIC) database also shown (below). **b** Violin plot shows sequencing error rate of human DNA, BRCAPTORs and error-corrected sequences at cancer-associated mutations (from the COSMIC database).
