# Supplementary material for: Library adaptors with integrated reference controls improve the accuracy and reliability of nanopore sequencing
Source: Nat Commun. 2022 Oct 28;13:6437. doi: 10.1038/s41467-022-34028-8 (PMC9616880; doi:10.1038/s41467-022-34028-8)
Supplement: Supplementary file 3 — Description of Additional Supplementary Information [file 41467_2022_34028_MOESM3_ESM.docx]

**Supplementary Data 1 Sequences of CAPTORS used in this project.** Table provides CAPTOR name (Column A) and the Complete CAPTOR sequences (Column B). Listed CAPTORS include both Forward (F) and Reverse (R) sequences. BRCAPTOR sequences are also listed on rows 152 – 154.
